# Etiology of Contagious Diseases

**Published:** 1888-01

**Authors:** J. W. McLaughlin

**Affiliations:** Austin, Texas


### THE ETIOLOGY OF INFECTIOUS DISEASES.

*By J. W. McLaughlin, M. D., Austin, Texas.*

(Being substantially his remarks in the discussion of two papers read at the recent meeting of the Austin District Medical Society.)

(Published in the Official Organ of the Society by vote of the Committee.)

THE papers which have just been read, and are now before this society for discussion, one upon Septic Fever, and the other upon Typhoid Fever, present some very important subjects for our consideration.

I will restrict my remarks to the etiology of infectious diseases, or the nature of contagion.

We have long known certain facts or phenomena of contagion, but had no intelligent knowledge of its nature until the "Compound Microscope" opened to our vision a microscopic vision of vegetable organisms, and the study of micro-botany taught us their marvelous properties.

Among the many theories of contagion which have been advocated at different times, but two seem worthy of much consideration; one is "Liebig's Theory," and the other the "Germ Theory."

According to Liebig, contagion, fermentation and putrefaction are caused by matter in a state of chemical change capable of imparting a similar change to other matter, by that unknown process, called "catalysis."

The "Germ Theory" assumes that these processes (fermentation, putrefaction and contagion) are caused by specific bacteria.

I will endeavor to show that both theories are partially correct: that the "Liebig Theory" when properly modified is complemental to the "Germ Theory." The known phenomena of a contagion may be summarised as follows:

1. The contagious principle, when suitably environed, will multiply and increase in amount.

2. It produces only its own kind as unerringly as do animals or vegetables.

3. It is portable; can be conveyed from place to place by infected individuals, clothing, food, etc.

4. It is destroyed by high temperatures and antiseptics.

5. Infectious diseases have a period of incubation, as a rule run a typical course, and one attack usually protects the individual from other invasions of the same disease.

6. The contagious principle can often be modified by "attenuation," and thus be made a protective agent against the virulent form.

A knowledge of these facts enables us to determine the value of the different theories of contagion. No theory can be accepted which is incompetent to rationally explain these pnenomena.

A moments reflection will convince us that this rule will exclude the old theory of polluted air, or of miasm, as well as the catalytic theory of Liebig, as propounded by its author!

I shall try to show that the "Germ Theory" when complemented by the "Liebig Theory" in its new form, offers a satisfactory explanation of these phenomena, and is sustained by other proofs equally as strong.

As contagion, infection, putrefaction and fermentation possess, in common, many points of resemblance, and result from a similar cause, it is necessary we should review some of the known laws governing these processes. A familiar illustration of fermentation is that which takes place in the brewer's vats.

It is known when yeast is added to the fermentable liquid called "Wort," that fermentation soon commences. The common products of this fermentation are "carbonic acid gas," "alcohol" and "yeast;" the first bubbles up through the liquid and escapes into the air; the second remains in solution, whilst the third, which had been originally added, is found to have increased ten-thousand-fold.

When a certain per cent. of alcohol is formed, fermentation is arrested and the further production of alcohol stopped; or if the required per cent. of alcohol be originally added to the wort, fermentation cannot be excited; if, however, the alcohol is removed, fermentation will begin and continue until the required per cent. is found.

From this we may infer that alcohol, a product of fermentation, will arrest this action when added in sufficient amount.

The "yeast," which has been so largely increased in amount, is deserving of our closest attention, for it is the active cause of fermentation, whilst a knowledge of its nature and methods of work throw great light upon the nature and working laws of contagion.

Yeast is not only the active cause of fermentation, but this action takes place only in immediate contact with this substance, as proven by the following experiment: Separate by a membranous diaphragm the "wort" contained in a vessel; on one side of this diaphragm place your yeast. Now, in accordance with the "laws of osmosis," the liquid wort and what it holds in solution, can readily pass from one side to the other through this diaphragm, whilst the solid yeast is confined to the side in which it was placed: under these circumstances fermentation occurs only on that side of the diaphragm which contains the yeast.

It may appear strange, but it is an important fact that alcohol does not always result from growing yeast in brewers wort; in fact, Oscar Brefield, by a peculiar artifice, succeeded in growing this without producing a particle of alcohol; a very significant fact, to which I shall again refer.

No doubt you are aware that yeast when viewed under a microscope of sufficient power, is found to be entirely composed of one celled vegetable organisms, or, if you please, bacteria.

It would prove very instructive and pertinent to our present inquiry to know how these yeast organisms or bacteria excite fermentation and produce alcohol. Many and various reasons have been offered in explanation. The one I shall give you to-day is in a measure, the result of my own reflections. It offers, I conceive, a rational explanation of these phenomena, and aspires to explain greater things, e. g., the relation of the individual to his physical environment, medical physics, including the power and potency of drugs, etc., none of which, however, concerns our present inquiry.

The science of chemistry is built upon the "Atomic Theory" which teaches that all matter can be reduced to its ultimate particles or atoms. That these atoms are indestructible and indivisible; are, in fact, the foundation rocks of the universe, and conform to the same laws, whether obtained from a meteoric rock, a messenger, perhaps, from some former planet, from the interior of the earth, or the air that surrounds it. Each and every atom has its equivalent of force, which gives it its distinctive properties, and this force is inseparable from the atom.

The Science of Physics teaches that force, like matter, is transmutable; e. g., as matter may be solid, in stone coal, liquid in water, or vapor in steam, so force may assume various forms, as latent sun heat of former periods, in coal, which can be changed into mechanical motion, and this again into electricity, heat, light, etc; in brief, force is molecular motion.

We thus have the ultimate particles of matter, atoms or molecules, possessed of distinctive vibrations or motions, which are capable of being acted upon by other vibrations or motions. This may be familiarly illustrated by what happens when sound waves of the same amplitude, and traveling toward each other, meet and produce silence, or when traveling in the same direction, if the one strikes the other properly, the sound will be increased; waves of water may be arrested or increased in the same way.

The facts I wish to impress, are:

1. Matter is composed of ultimate particles.
2. These are in constant motion.
3. Other atoms in particles act upon them by increasing or decreasing this movement as the periods of recurrence in the molecule of the one substance may or may not be in accord with that of the other. Now, iron, like other matter, is composed of atoms in constant motion; heat is a mode of molecular motion which increases the swing of the iron atoms, hence, as the iron becomes heated it increases in bulk, and, as its atoms are driven further and further apart by the heat waves. Just as the breath blown against a receding pendulum will increase its swing. The iron becomes liquid, and finally the atoms are driven beyond their attractive affinities, and it becomes a gas. The law of molecular activities is universal, and through this I shall attempt an explanation of how the yeast bacteria excite fermentation and produce alcohol. The alcohol and carbonic acid which is formed, contain the same atoms in the same proportion, but differently arranged, as did the sugar which was destroyed. In other words, the atoms of the sugar contained in the wort are torn asunder and liberated from their attractions. These atoms then recombine to form alcohol and carbonic acid, which are simpler compounds than sugar. We know that the yeast organisms do this work. I offer the following explanation of the manner and methods by which this is done. The molecular movements of the yeast bacteria are so timed in their periods of recurrence that they increase those of the sugar and swing them beyond their attractions, and thus allow to form new and simpler compounds in accordance with known chemical laws.

I do not claim that all fermentation is caused by bacteria, but I do claim, whatever the cause may be, it acts in this manner, and this theory offers a rational explanation of the hitherto unknown process of catalysis.

Let us now examine how perfectly these facts regarding fermentation, explain the phenomena of contagion, assuming that contagion is caused by specific bacteria.

At the outset let it be known that one kind or variety of bacteria is not able to produce all kinds of infection. As the yeast bacteria can excite only the vinous fermentation, and other fermentations are excited by micro organisms peculiar to each, so, different infectious diseases are caused by different infectious organisms. When the organism of an infectious blood disease has gained entrance into the blood of an individual, it finds in this fluid the conditions necessary to its growth by reproduction.

The contagious principle will thus continue to increase in the blood until this process is arrested.

Inasmuch as these bacteria breed true, that is, produce always their own kind, the diseases they produce observe the same law.

As these organisms are entities, although exceedingly small ones, it is easily understood, how they may be conveyed from place to place, in various ways, and thus explain, how contagion is similarly transmitted.

High temperatures and anti-septics destroy them in the same manner, and according to the same laws, by which contagion is thus destroyed.

You will readily conceive that those phenomena of contagion, embraced in laws fifth or sixth, to the effect that contagious diseases, as a rule, observe a period of incubation, run a typical course, do not occur twice in the same individual, and may sometimes be prevented by inoculation, or vaccination with the modified poison, are least understood, and most difficult of explanation; hence, I ask your patience, whilst I endeavor to make these matters clear.

I must again remind you of what occurs in vinous fermentation, for we are reasoning by analogy. On the one hand, we have sugar in solution, or molecular division; these sugar molecules are composed of smaller bodies, or atoms, held together by attractive and repellent forces, which keep them in constant motion; remove these atoms beyond their attractions and the sugar is destroyed, whilst the atoms again combine in simpler compounds, *e. g.*, alcohol and carbonic acid; on the other hand, we have the yeast bacteria, also composed of molecules and atoms; the motions of these are so timed in their periods of recurrence, that they swing the atoms of sugar beyond their attractions, and thus disrupt it.

In the light furnished by these facts, let us inquire what takes place when bacteria grow in living blood.

It is evident, that such growth and increase is at the expense of certain nutritive material which the blood contains; if the blood did not contain this substance, the bacteria could not live in it.

It is also a reasonable supposition, and in harmony with analogy, that the bacteria, because of their molecular movements, excite in the blood a species of fermentation;—shake apart certain molecules which it contains, and by thus liberating their atoms, permit them to re-unite, and form other and simple compounds.

Now, it is a matter of fact, that such compounds are formed in the blood, and are termed ptomaines, or leucomanes. These substances are poisons; give rise to fever and other clinical symptoms peculiar to the special disease.

If infectious bactera produce ptomaines and leucomanes, and these poisons produce disease, it is proper to inquire, and no doubt you have mentally done so, how it is possible for a person with an infectious fever to recover; if these poisons are formed in this way, will not this process continue till the victim expires?

The answer to this inquiry at first may seem contradictory, and paradoxical; but I trust upon further investigation, you will be satisfied it is correct.

The alcohol produced by yeast bacteria is a poison to these organisms, and when a sufficient amount of alcohol is furnished, the action of these bacteria becomes at once arrested. This action embodies a principle of general application, and is exemplified by what occurs when an individual is hermetically closed in a room; it is known that carbonic acid, a product of respiration, and hence of the individual, is poisonous, and when supplied in sufficient amount, will destroy his life.

In harmony with this law, and in the light of analogy, we claim that infectious bacteria, like yeast bacteria, furnish their own poisons,—ptomaines and leucomanes,—that these in sufficient amount, arrest the bacterial action, or destroy the bacteria. Were it not for this law, all infectious diseases would necessarily result in the death of the individual.

This is a new field of inquiry, and I appreciate the wisdom of going slow in its investigation; the explanation which I have submitted, may or may not be true; if not true, there is no truth in analogy. The incubative stage of infectious diseases, according to the "germ theory," corresponds with that time between the introduction of the poisonous bacteria and their sufficient increase to produce ptomaines; in fact, it is these latter substances, and not the bacteria, that poison the blood, and give rise to the clinical phenomena of the disease. As infectious bacteria feed upon, and perhaps destroy certain nutritive material in the blood, it is readily seen that this would protect the individual from a second invasion, until this material is reformed ; if never reformed, this protection would be perpetual.

If these infectious bacteria could, by any means, have their distinctive molecular movements, so changed that their periods of recurrence would not be in unison with, and hence could not shake apart certain molecules in the blood, in other words, could not produce the poisonous ptomaines, and all this occur without destroying their power of reproduction and their habit of feeding upon certain nutritive blood material, we would have in these modified bacteria, a protective means against the infectious disease. Fortunately this has been done in a few cases, e. g. anthrax, [illegible]en cholera, and perhaps in hydrophobia and yellow fever.

The experiments of Oscar Brefield, of which I have spoken, have no doubt occured to you in this connection. You remember, he was enabled by an ingenious artifice to so modify yeast bacteria, that they would grow and increase by reproduction in brewers' wort, without producing a particle of alcohol.

It is at once apparant to all, that this is an analogous process ; the alcohol represents the ptomaines produced by the bacteria.

As the yeast bacteria can be so modified, it is reasonable to suppose that the same can be done with septic bacteria. We are not compelled to rely upon analogy to establish this principle, for it is known that passing small-pox virus through the cow gives it this modification ; subjecting the bacteria of anthrax, chicken cholera and swine plague, to certain methods of treatment, render them unable to produce ptomaines, and thus make them protective against the diseases produced by the unmodified microbes.

Liebig's theory, as taught by its author, contains many defects. The short time at my disposal, is not sufficient to point them out.

When, however, you substitute a living germ, possessing certain molecular activities, with the power of breeding true, for Liebig's "matter undergoing chemical change," all difficulties disappear, and all defects are remedied.

There are other and stronger proofs of the "germ theory;" those furnished by innoculation experiments with pure cultures of infectious bacteria, which I would take pleasure in explaining, and especially in refuting the arguments of those who regard bacteria, as the result, rather than the cause of disease, if I was not warned that my time is up, and your patience is exhausted.

